# Efficacy and Safety of Supramolecular Salicylic Acid Peeling Combined With Intense Pulsed Light Versus Intense Pulsed Light Monotherapy in Erythematotelangiectatic‐Predominant Rosacea: A Retrospective Real‐World Comparative Study

**DOI:** 10.1111/jocd.71249

**Published:** 2026-10-16

**Authors:** Birao Fan, Ruixing Yu

**Affiliations:** ^1^ Department of Dermatology China‐Japan Friendship Hospital Beijing China

**Keywords:** adverse events, erythematotelangiectatic‐predominant rosacea, intense pulsed light, real‐world study, rosacea, supramolecular salicylic acid, VISIA

## Abstract

**Background:**

Evidence is limited on whether supramolecular salicylic acid (SSA) peeling adds benefit to intense pulsed light (IPL) for erythematotelangiectatic‐predominant rosacea in routine practice. We compared real‐world effectiveness and short‐term safety of SSA + IPL versus IPL monotherapy using objective imaging and patient‐centered outcomes.

**Methods:**

In this single‐center retrospective cohort, consecutive adults with erythematotelangiectatic‐predominant rosacea treated from January 2022 to December 2025 received SSA + IPL or IPL alone according to routine‐care preference. Outcomes were assessed at baseline and 3 months after the final session. Objective measures included VISIA/ImageJ‐derived erythema and hyperpigmentation metrics, as well as VISIA wrinkle and pore indices. Clinical and patient‐reported outcomes included Clinician's Erythema Assessment (CEA), flushing severity, stinging numerical rating scale (NRS), Rosacea Quality of Life (RosaQoL), and Global Aesthetic Improvement Scale (GAIS). Treatment‐related adverse events (AEs) within 1 week after the final session were collected via a mobile questionnaire using a modified CTCAE‐like 0–3 grading.

**Results:**

Fifty‐seven patients were analyzed (SSA + IPL, *n* = 25; IPL, *n* = 32). Both regimens improved objective imaging metrics and clinical outcomes at 3 months. Compared with IPL alone, SSA + IPL was associated with a greater improvement in erythema area than IPL monotherapy, and this association remained significant after adjustment for age. In contrast, the between‐group difference in erythema concentration was attenuated after age adjustment. Greater improvements in hyperpigmentation‐related measures, pore index, RosaQoL, and GAIS were also observed with SSA + IPL, whereas differences in several clinician‐ and patient‐reported symptom measures were not statistically robust.

**Conclusions:**

In this retrospective real‐world cohort, SSA + IPL was associated with greater improvement in erythema area and selected secondary outcomes than IPL monotherapy, although not all between‐group differences remained robust after adjustment. These findings should be considered hypothesis‐generating and require confirmation in adequately powered prospective controlled studies.

## 1 Introduction

Rosacea is a chronic, relapsing inflammatory dermatosis predominantly affecting the central face and frequently characterized by persistent erythema, flushing, and telangiectasia, which collectively impose substantial psychosocial burden and trigger‐avoidance behaviors. Current consensus increasingly favors a phenotype‐based framework, in which persistent centrofacial erythema and visible telangiectasia define a distinct therapeutic target that may not be adequately controlled by anti‐inflammatory pharmacotherapy alone [1, 2, 3]. Clinical guidelines therefore recognize a role for light‐ and laser‐based devices when vascular phenotypes predominate or when residual erythema persists despite appropriate skincare and trigger management [1, 3].

Intense pulsed light (IPL) is widely applied for vascular facial indications because its broad spectrum can be tailored to target oxyhemoglobin while simultaneously addressing dyschromia and textural concerns. Early clinical studies reported meaningful reductions in rosacea‐associated erythema and telangiectasia after repeated IPL sessions [4, 5]. More recently, an IPL‐focused systematic review concluded that most studies demonstrate improvement in erythema/telangiectasia with predominantly transient adverse effects, while also highlighting substantial heterogeneity in parameters, outcome measures, and study quality [6]. In parallel, a meta‐analysis published in Journal of Dermatological Treatment compared pulsed dye laser and IPL for facial erythema of rosacea and suggested both modalities can be effective, underscoring the need for pragmatic, real‐world evidence with objective outcome metrics [7]. Importantly, treatment goals in rosacea are closely linked to patient‐perceived “clearance” and satisfaction, supporting the inclusion of both clinician‐ and patient‐reported end points rather than relying on a single scale [8].

Chemical peeling is often incorporated into aesthetic practice to improve epidermal turnover and post‐inflammatory dyschromia. Supramolecular salicylic acid (SSA), designed to enhance tolerability compared with conventional salicylic acid, has been reported to improve rosacea‐related symptoms and skin barrier–associated parameters in clinical studies, and combination strategies with vascular lasers have also been explored [9, 10]. However, whether SSA provides incremental benefit beyond IPL alone for vascular‐predominant rosacea remains unclear.

Therefore, we conducted a real‐world study to evaluate the effectiveness and safety of SSA plus IPL compared with IPL alone for ETR‐predominant rosacea. Outcomes were assessed using standardized VISIA‐derived objective indices complemented by clinician‐ and patient‐reported scales, with durability evaluated at 3 months after the final treatment and short‐term tolerability captured within 1 week post‐treatment.

## 2 Materials and Methods

### 2.1 Study Design and Ethics

This single‐center, retrospective, real‐world comparative study was conducted at the Department of Dermatology, China‐Japan Friendship Hospital. Consecutive patients with ETR‐predominant rosacea treated between January 2022 and December 2025 were screened.

### 2.2 Participants

Consecutive adults aged 18–65 years with ETR‐predominant rosacea were screened. ETR‐predominant rosacea was defined by persistent centrofacial erythema, with or without telangiectasia, as the dominant phenotype and primary treatment target. Mild concomitant inflammatory papules were permitted and recorded as baseline phenotypic variables. Exclusion criteria were papulopustular‐predominant rosacea, baseline pustules, severe inflammatory flares, acne vulgaris‐dominant lesions, other facial dermatoses affecting erythema assessment, incomplete baseline data, pregnancy or lactation, irregular or < 3‐month oral tetracycline or topical therapy, and facial energy‐based procedures within the preceding 3 months. Patients receiving irregular background rosacea therapies were excluded to minimize confounding from uncontrolled anti‐inflammatory treatment. Eligible patients received SSA + IPL or IPL monotherapy according to patient preference. Patients with incomplete clinical data or irregular follow‐up were excluded from the final analysis (Figure S1). The complete eligibility criteria are summarized in Table S1.

### 2.3 Treatment Protocols

All procedures were performed by experienced dermatologists. In the combination group, 30% SSA was applied after facial cleansing, with petrolatum applied to the periorbital area, nasal alae, lip margins, and other areas with visible barrier disruption for protection. SSA was removed at the clinical endpoint and the skin was thoroughly rinsed and dried. IPL was initiated immediately thereafter, typically within approximately 5 min after complete SSA removal using the Lumenis M22 system; the monotherapy group received IPL without prior peeling. Parameters were individualized according to Fitzpatrick skin type and erythema pattern within a standardized vascular‐treatment framework summarized in Supporting Information and Table S2. Coupling gel and contact cooling were used throughout. Treatments were performed every 4–6 weeks for three sessions. After the third session, an additional session could be considered if clinically meaningful residual erythema or telangiectasia remained and both the treating dermatologist and patient considered further treatment necessary. In the present cohort, all patients were considered to have achieved satisfactory improvement after three sessions, and no patient underwent a fourth session.

### 2.4 Outcome Assessments and Follow‐Up Schedule

Outcomes were assessed at baseline and 3 months after the final treatment. No maintenance pharmacotherapy was administered during follow‐up, and patients were advised to continue trigger avoidance, gentle skincare, and photoprotection. Specific cleansers, moisturizers, and sunscreen products were not standardized.

Standardized VISIA imaging was used to assess erythema and hyperpigmentation. Cross‐polarized Red Areas images and ultraviolet images were quantified using ImageJ with “Limit to Threshold” enabled; affected area and mean gray value were recorded. VISIA‐generated wrinkle and pore indices were also collected. In the VISIA analysis system, wrinkles are identified as visible furrows, folds, or creases in the skin, whereas pores are identified as darker circular surface openings based on their contrast and morphology. The corresponding quantitative VISIA outputs were used as texture‐related exploratory measures. For interpretative purposes, erythema‐related VISIA measures and CEA were considered key disease‐oriented outcomes, whereas pigmentation‐ and texture‐related imaging measures were considered exploratory outcomes. Patient‐reported measures were considered supportive outcomes. Because of the retrospective design, no single primary endpoint was prospectively prespecified.

Clinical and patient‐reported outcomes included CEA, self‐assessed facial redness, flushing severity, stinging NRS, RosaQoL, and GAIS. RosaQoL was calculated as the mean of its 21 item scores, yielding a score from 1 to 5, with higher scores indicating greater quality‐of‐life impairment. GAIS was assessed on a 5‐point ordinal scale: 1, worse; 2, no change; 3, improved; 4, much improved; and 5, very much improved. At the 3‐month assessment, no patient was rated as worse or unchanged; therefore, the observed GAIS categories in this cohort were 3–5.

For study‐level clinician assessments, standardized baseline and follow‐up images were anonymized, presented in randomized order, and stripped of treatment‐group and assessment‐time information. CEA scores used in the study analyses were assigned by the dermatologists on the basis of these blinded image assessments. Thus, although CEA was routinely recorded during clinical visits, the CEA values used for the present study were derived from the blinded study‐level reassessment rather than from unblinded routine‐care records.

To assess the reproducibility of the ImageJ‐based measurements, a randomly selected subset of 20 patients was independently evaluated by two dermatologists blinded to treatment allocation and assessment time point (Table S3). Baseline and follow‐up images were anonymized and presented in random order. Each ImageJ measurement was performed three times and averaged. Accordingly, 40 image sets (baseline and 3‐month follow‐up images from 20 patients) were included in the reliability analysis. Both assessors repeated the complete measurements after an interval of at least 2 weeks. Inter‐ and intra‐rater reliability was assessed using intraclass correlation coefficients (ICCs) with 95% confidence intervals. Inter‐rater reliability was evaluated using a two‐way random‐effects, absolute‐agreement ICC, whereas intra‐rater reliability was evaluated using a two‐way mixed‐effects, absolute‐agreement ICC.

### 2.5 Safety Assessment

Treatment‐related AEs within 1 week after the final session were collected using a mobile patient questionnaire. Erythema, edema, pain, dryness, stinging, pustules, exudation/crust, and hyperpigmentation were graded from 0 (none) to 3 (severe); incidence was defined as a score > 0, and incidence and ordinal severity were analyzed separately.

### 2.6 Statistical Analysis

Analyses were performed using SPSS version 30.0. Continuous variables are presented as mean (SD) or median (IQR), and categorical variables as *n* (%). Baseline characteristics were compared using Welch's *t*‐test, Fisher's exact test, Monte Carlo exact test, or Mann–Whitney *U* test, as appropriate. Within‐group changes were assessed using the Wilcoxon signed‐rank test, and improvement values were compared between groups using the Mann–Whitney *U* test. Given the significant baseline age difference between treatment groups, additional age‐adjusted analyses were performed using multivariable regression models, with improvement in each outcome as the dependent variable and treatment group and age as independent variables. For GAIS, an ordinal logistic regression model including treatment group and age was used. Adjusted between‐group estimates with 95% confidence intervals and *p* values were reported. To assess whether concomitant mild inflammatory papules influenced the findings, a sensitivity analysis was additionally performed after excluding all patients with baseline papules. AE incidence and severity were compared using Fisher's exact test and the Mann–Whitney *U* test, respectively. Complete‐case analyses were performed without imputation. Two‐sided *p* < 0.05 was considered statistically significant. Benjamini–Hochberg FDR adjustment was applied as a sensitivity analysis to efficacy outcomes, AE incidence outcomes, and AE severity outcomes as separate comparison families.

## 3 Results

### 3.1 Baseline Characteristics

Among 104 eligible patients who selected a treatment strategy, 57 (54.8%) had complete clinical and 3‐month follow‐up data and were included in the final analysis (SSA + IPL, *n* = 25; IPL, *n* = 32). Reasons for post‐selection exclusion are detailed in Figure S1. The cohort was predominantly female, with Fitzpatrick skin Type III accounting for most cases. The SSA + IPL group was older than the IPL group (36.5 ± 4.58 vs. 31.69 ± 4.35 years, *p* < 0.001). Other baseline demographic and phenotypic characteristics, including sex, Fitzpatrick skin type, prior treatment, baseline erythema area and concentration, CEA, self‐assessed facial redness, flushing severity, stinging NRS, RosaQoL, and baseline papular involvement, were broadly comparable between groups. Baseline papules were observed in 5 patients (20.0%) in the SSA + IPL group and 7 patients (21.9%) in the IPL group, whereas no baseline pustules were observed in either group (Table 1).

**TABLE 1:** Baseline demographic and phenotypic characteristics of patients with ETR‐predominant rosacea.

	SSA + IPL	IPL	*p*
Patient no.	25	32	
Gender, *n* (%)			1.000
Male	4 (16.0)	6 (18.8)	
Female	21 (84.0)	26 (81.3)	
Age, years, mean (SD)	36.5 (4.58)	31.69 (4.35)	< 0.001
Fitzpatrick skin type, *n* (%)			0.485
Type III	20 (80.0)	28 (87.5)	
Type IV	5 (20.0)	4 (12.5)	
Prior treatment			0.341
None	8 (32.0)	15 (46.9)	
Minocycline	13 (52.0)	14 (43.8)	
IPL	2 (8.0)	0	
SSA	2 (8.0)	3 (9.4)	
Number of treatment sessions			1.000
3	19 (76.0)	25 (78.1)	
4	6 (24.0)	7 (21.9)	
Baseline erythema area (Median [IQR], mm^2^)	2295.59 (2093.97, 2446.55)	2358.09 (2079.50, 2492.80)	0.968
Baseline erythema concentration (Median [IQR], %)	11.98 (11.06, 13.46)	12.15 (10.64, 13.27)	0.694
Baseline CEA, median (IQR)	3 (3, 4)	3 (3, 4)	0.676
Baseline self‐assessed facial redness, median (IQR)	3 (3, 4)	3 (3, 4)	0.731
Baseline flushing severity, median (IQR)	2 (2, 3)	2.5 (2, 3)	0.077
Baseline stinging NRS, median (IQR)	3 (3, 4)	3 (3, 4)	0.747
Baseline RosaQoL, median (IQR)	4.2 (3.6, 4.5)	4.2 (3.9, 4.4)	0.375
Patients with baseline papules, *n* (%)	5 (20.0)	7 (21.9)	1.000
Total baseline papule count, n	121	193	Descriptive only
Baseline papule count per patient, median (IQR)	0 (0, 0)	0 (0, 0)	0.787
Patients with baseline pustules, *n* (%)	0 (0.0)	0 (0.0)	NA
Baseline pustule count per patient, median (IQR)	0 (0, 0)	0 (0, 0)	NA

*Note:* *p* values were calculated using Fisher's exact test for binary categorical variables, Monte Carlo exact test for multi‐category categorical variables, Welch's *t*‐test for age, and Mann–Whitney *U* test for ordinal or non‐normally distributed continuous variables. Total baseline papule count was presented descriptively and was not subjected to statistical testing because it was not a patient‐level independent variable.

### 3.2 VISIA Imaging Outcomes

At 3 months after the final session, both regimens produced significant within‐group improvements in objective VISIA metrics, including erythema area and erythema concentration (all *p* < 0.001, Table 2). In the unadjusted analyses, SSA + IPL was associated with greater improvement than IPL monotherapy in both erythema area (*p* < 0.001) and erythema concentration (*p* = 0.015). After adjustment for age, the between‐group difference remained significant for erythema area (adjusted difference, 385.66 mm^2^; 95% CI: 243.92–527.39; *p* < 0.001), whereas the difference in erythema concentration was attenuated and no longer statistically significant (adjusted difference, −0.06 percentage points; 95% CI: −0.45 to 0.34; *p* = 0.777).

**TABLE 2:** Objective VISIA‐based quantitative changes following SSA + IPL versus IPL monotherapy in patients with erythematotelangiectatic‐predominant rosacea.

	Current treatment	Baseline	After treatment	Improvement	*p* ^1^	*p* ^2^
Erythema (Median [IQR], mm^2^)	SSA + IPL	2295.59 (2093.97, 2446.55)	1058.89 (938.58, 1180.09)	1182 (1034.51, 1484.24)	< 0.001	< 0.001
	IPL	2358.09 (2079.50, 2492.80)	1551.82 (1297.32, 1701.08)	804.07 (771.57, 815.57)	< 0.001	
Erythema concentration (Median [IQR], %)	SSA + IPL	11.98 (11.06, 13.46)	9.81 (8.83, 11.57)	2.20 (2.00, 2.26)	< 0.001	0.015
	IPL	12.15 (10.64, 13.27)	9.74 (8.76, 10.80)	1.87 (1.75, 2.19)	< 0.001	
Hyperpigmentation (Median [IQR], mm^2^)	SSA + IPL	491.72 (381.83, 612.05)	269.36 (180.39, 383.72)	215.11 (193.38, 245.58)	< 0.001	< 0.001
	IPL	495.74 (368.66, 567.59)	384.11 (294.24, 447.80)	113.19 (102.08, 130.96)	< 0.001	
Hyperpigmentation concentration (Median [IQR], %)	SSA + IPL	7.94 (7.31, 8.53)	5.72 (5.22, 6.41)	2.10 (1.96, 2.18)	< 0.001	< 0.001
	IPL	7.90 (7.46, 8.36)	6.39 (5.95, 6.79)	1.60 (1.33, 1.75)	< 0.001	
Pore index, Median [IQR]	SSA + IPL	445 (325.00, 476.00)	379 (253.00, 402.00)	69 (65, 80)	< 0.001	< 0.001
	IPL	441 (377.5, 483.5)	412.50 (347.25, 458.75)	30.5 (24.50, 34.00)	0.002	

*Note:* Unadjusted *p* values are shown. *p* ^1^, within‐group comparison by Wilcoxon signed‐rank test; *p* ^2^, between‐group comparison by Mann–Whitney *U* test. FDR‐adjusted *Q* values were calculated as a sensitivity analysis and are presented in Table S2.

Exploratory hyperpigmentation‐related imaging outcomes also improved significantly in both groups. After adjustment for age, greater improvements remained associated with SSA + IPL for hyperpigmentation area (adjusted difference, 115.43 mm^2^; 95% CI: 85.02–145.84; *p* < 0.001) and hyperpigmentation concentration (adjusted difference, 0.69 percentage points; 95% CI: 0.36–1.03; *p* < 0.001). For texture‐related indices, the adjusted between‐group difference remained significant for pore index (adjusted difference, 43.01; 95% CI: 36.60–49.42; *p* < 0.001), whereas no significant difference was observed for wrinkle index (adjusted difference, 2.32; 95% CI: −16.38 to 21.01; *p* = 0.805; Table S4).

The principal findings for erythema area, hyperpigmentation‐related measures, and pore index also remained significant after FDR adjustment (Table S5). Representative clinical photographs and VISIA maps are shown in Figure 1.

**FIGURE 1:**
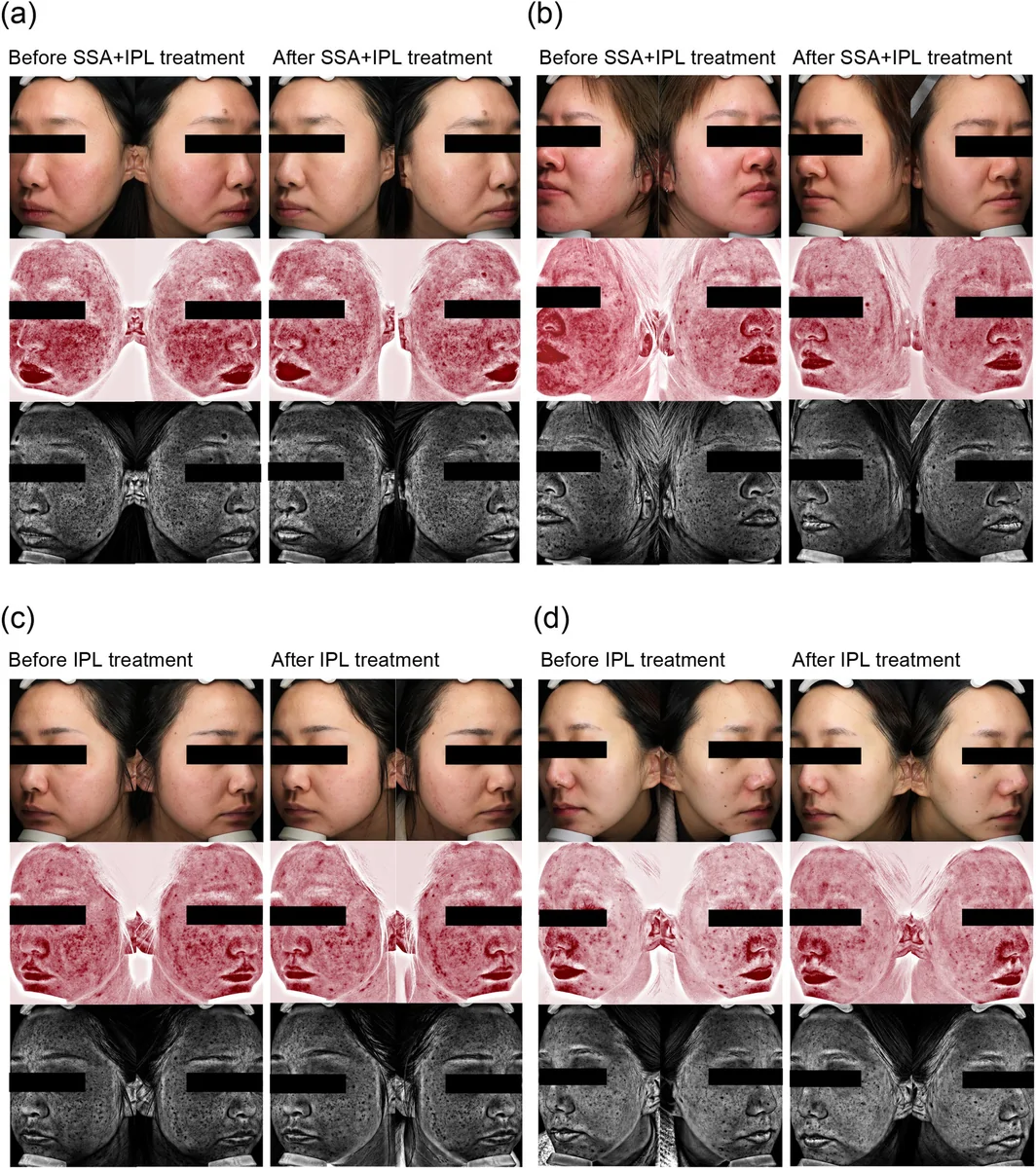
Representative clinical photographs and VISIA imaging maps before and 3 months after the final treatment session in patients with ETR‐predominant rosacea. Representative cases are shown for the SSA + IPL group (a, b) and the IPL monotherapy group (c, d). Patients had persistent centrofacial erythema as the dominant phenotype; mild inflammatory papules, if present at baseline, were recorded as phenotypic variables but were not the primary therapeutic target. No baseline pustules were observed in either group. From top to bottom, the rows show standard facial photographs, VISIA cross‐polarized “Red Areas” images, and VISIA ultraviolet images. The eye region was masked to protect patient privacy. ETR‐predominant rosacea, erythematotelangiectatic‐predominant rosacea; IPL, intense pulsed light; SSA, supramolecular salicylic acid.

In a sensitivity analysis excluding all 12 patients with baseline papules, 45 patients remained (SSA + IPL, *n* = 20; IPL, *n* = 25). The between‐group difference remained significant for erythema area improvement (median improvement, 1180.35 vs. 779.97 mm^2^; *p* < 0.001), whereas the difference in erythema concentration was no longer statistically significant (2.215 vs. 1.920 percentage points; *p* = 0.059). Greater improvements with SSA + IPL also remained evident for hyperpigmentation area, hyperpigmentation concentration, pore index, and RosaQoL (all *p* < 0.001). These findings support the robustness of the principal imaging findings, particularly the between‐group difference in erythema area, after exclusion of patients with mild baseline papules (Table S6).

ImageJ‐based measurements showed excellent reproducibility. Inter‐rater ICCs ranged from 0.924 to 0.943 across the four erythema and hyperpigmentation parameters, while intra‐rater ICCs ranged from 0.947 to 0.980 across both assessors (Table S3).

### 3.3 Clinical Effectiveness

Clinician‐ and patient‐reported outcomes improved significantly from baseline in both groups (all *p* < 0.001, Table 3). In unadjusted between‐group analyses, SSA + IPL was associated with greater improvement in RosaQoL (*p* < 0.001), with nominally greater improvements also observed for CEA and stinging NRS. After adjustment for age, the between‐group difference remained significant for RosaQoL (adjusted difference, 0.79; 95% CI: 0.54–1.03; *p* < 0.001). In contrast, adjusted differences were not statistically significant for CEA (adjusted difference, 0.34; 95% CI: −0.16 to 0.83; *p* = 0.184) or flushing severity (adjusted difference, −0.11; 95% CI: −0.60 to 0.38; *p* = 0.657).

**TABLE 3:** Physician‐ and Patient‐Reported Clinical Outcomes After Treatment in Patients With ETR‐Predominant Rosacea.

	Current treatment	Baseline	After treatment	Improvement	*p* ^1^	*p* ^2^
CEA, median [IQR]	SSA + IPL	3 (3, 4)	0 (0, 1)	3 (2, 3)	< 0.001	0.040
	IPL	3 (3, 4)	1 (0,1)	2.5 (2, 3)	< 0.001	
Subject Self‐Assessment for rosacea facial redness, median [IQR]	SSA + IPL	3 (3, 4)	0 (0, 1)	3 (2, 3)	< 0.001	0.108
	IPL	3 (3, 4)	1 (0, 1)	3 (2, 3)	< 0.001	
RosaQoL, median [IQR]	SSA + IPL	4.2 (3.6, 4.5)	1.5 (1.3, 1.8)	2.4 (2.1, 3.1)	< 0.001	< 0.001
	IPL	4.2 (3.9, 4.4)	2.4 (2.2, 2.7)	1.7 (1.6, 1.8)	< 0.001	
Flushing severity, median [IQR]	SSA + IPL	2 (2, 3)	0 (0, 1)	2 (1, 2)	< 0.001	0.234
	IPL	2.5 (2, 3)	0 (0, 1)	2 (1, 3)	< 0.001	
Stinging NRS, median [IQR]	SSA + IPL	3 (3, 4)	1 (1, 1)	2 (2, 3)	< 0.001	0.052
	IPL	3 (3, 4)	1 (1, 1)	2 (1, 2)	< 0.001	

*Note:* Unadjusted *p* values are shown. *p* ^1^, within‐group comparison by Wilcoxon signed‐rank test; *p* ^2^, between‐group comparison by Mann–Whitney *U* test. FDR‐adjusted *Q* values were calculated as a sensitivity analysis and are presented in Table S2.

Nominal age‐adjusted differences were observed for self‐assessed facial redness (adjusted difference, 0.47; 95% CI: 0.03–0.91; *p* = 0.039) and stinging NRS (adjusted difference, 0.62; 95% CI: 0.01–1.22; *p* = 0.045); however, neither remained significant after FDR adjustment, and these findings should therefore be interpreted cautiously.

GAIS distributions favored SSA + IPL (Figure 2). In age‐adjusted ordinal logistic regression, SSA + IPL was associated with higher odds of being in a better GAIS category than IPL monotherapy (OR, 29.00; 95% CI: 6.34–132.58; *p* < 0.001), and this association remained significant after FDR adjustment (Tables S4 and S5).

**FIGURE 2:**
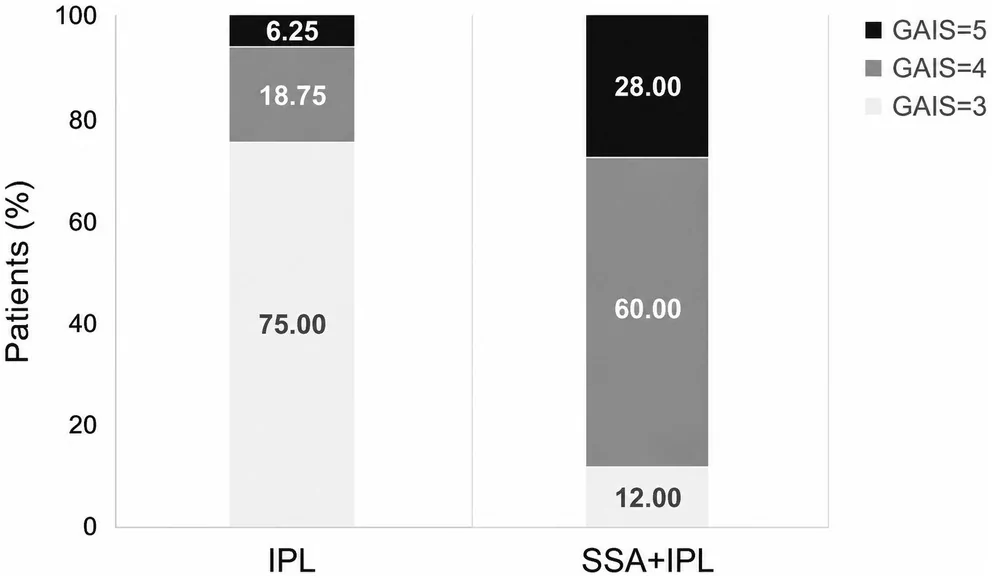
Distribution of Global Aesthetic Improvement Scale (GAIS) ratings at 3 months after the final treatment session in the SSA + IPL and IPL groups. Stacked bars show the percentage of patients in each GAIS category (GAIS = 3, improved; GAIS = 4, much improved; GAIS = 5, very much improved) at the 3‐month posttreatment assessment. IPL group, *n* = 32; SSA + IPL group, *n* = 25. Values displayed within bars represent percentages. The distribution of GAIS categories differed between groups (*p* < 0.001).

### 3.4 Patient‐Reported Treatment‐Related Adverse Events

Most treatment‐related AEs were mild or moderate and transient, with no severe events reported (Table 4). The incidence of all recorded AEs was comparable between groups. In ordinal severity analyses, edema was more pronounced after SSA + IPL than after IPL monotherapy (median [IQR], 2 [1–2] vs. 1 [1–1]; unadjusted *p* = 0.009), whereas stinging severity showed a nonsignificant numerical difference (*p* = 0.075). Neither severity finding remained statistically significant after FDR adjustment.

**TABLE 4:** Treatment‐related adverse events within 1 week after therapy in the SSA + IPL and IPL groups.

Adverse reactions	Current treatment	Count, *n* (%)	Score, median (IQR)	*p* ^1^	*p* ^2^
Erythema	SSA + IPL	25 (100.0)	1 (1, 2)	1.000	0.071
	IPL	32 (100.0)	1 (1, 1)		
Edema	SSA + IPL	25 (100.0)	2 (1, 2)	1.000	0.009
	IPL	32 (100.0)	1 (1, 1)		
Pain	SSA + IPL	12 (48.0)	0 (0, 1)	0.599	0.546
	IPL	18 (56.3)	1 (0, 1)		
Dryness	SSA + IPL	15 (60.0)	1 (0, 1)	1.000	0.856
	IPL	20 (62.5)	1 (0, 1)		
Stinging	SSA + IPL	18 (72.0)	1 (0, 2)	0.775	0.075
	IPL	21 (65.6)	1 (0, 1)		
Pustules	SSA + IPL	9 (36.0)	1 (0, 1)	0.575	0.536
	IPL	9 (28.1)	0 (0, 1)		
Exudation and crust	SSA + IPL	11 (44.0)	0 (0, 1)	0.410	0.331
	IPL	10 (31.3)	0 (0, 1)		
Hyperpigmentation	SSA + IPL	14 (56.0)	1 (0, 1)	0.293	0.257
	IPL	13 (40.6)	0 (0, 1)		

*Note:* Severity was graded on a modified CTCAE‐like 0–3 scale: 0, none; 1, mild; 2, moderate; and 3, severe. AE incidence was defined as a score > 0. *p* ^1^ values for incidence were calculated using Fisher's exact test, and *p* ^2^ values for severity were calculated using the Mann–Whitney *U* test using the full ordinal score distributions. Unadjusted *p* values are shown; FDR‐adjusted *Q* values for incidence and severity analyses are presented separately in Table S2.

## 4 Discussion

In this real‐world cohort of patients with ETR‐predominant rosacea, both IPL monotherapy and SSA + IPL combination therapy were associated with improvements across objective imaging and clinical outcomes. The most consistent between‐group finding was a greater improvement in erythema area with SSA + IPL, which remained significant after adjustment for age and after exclusion of patients with baseline papules. In contrast, the between‐group difference in erythema concentration observed in the unadjusted analysis was not maintained in these sensitivity analyses. Greater improvements in hyperpigmentation‐related measures, pore index, RosaQoL, and GAIS were also observed with SSA + IPL, whereas several clinician‐ and patient‐reported symptom outcomes were less robust. Overall AE incidence was comparable between groups, although exploratory severity analyses suggested greater transient procedural irritation after combination treatment.

Rosacea is a chronic inflammatory dermatosis, and the ETR‐predominant phenotype is characterized by persistent centrofacial erythema and telangiectasia, often accompanied by burning, stinging, and reduced quality of life. Contemporary guidance emphasizes phenotype‐based management and the need to address both vascular and inflammatory components while minimizing triggers and barrier disruption [1, 2, 3]. Light‐based devices, including IPL and pulsed dye laser (PDL), are widely used for the erythema/telangiectasia phenotype, with evidence supporting reductions in erythema severity and telangiectasia burden [4, 5, 6]. Real‐world retrospective data also support the routine use of light/laser devices across rosacea subtypes [11]. Meta‐analytic data further indicate that IPL can significantly improve rosacea‐related erythema and patient‐reported severity, though treatment parameters and response magnitude vary across studies [6, 7]. In a pooled analysis, achieving “clear” or “almost clear” status was associated with patient‐relevant benefits [8], aligning with our inclusion of clinician scales, patient‐reported outcomes, and GAIS.

We acknowledge that salicylic acid peeling is not a conventional first‐line treatment for a strictly vascular or biologically “pure” ETR phenotype. Because rosacea phenotypes often overlap in routine practice, our cohort is more accurately described as ETR‐predominant rosacea, in which persistent centrofacial erythema was the leading manifestation and treatment target, while mild papular inflammatory lesions could coexist in a minority of patients. Accordingly, our findings should not be interpreted as evidence that SSA monotherapy directly treats isolated vascular erythema, but rather as exploratory evidence that adjunctive SSA may have practical value when combined with IPL in selected patients with ETR‐predominant rosacea.

A key question raised by our data is why SSA + IPL was associated with greater improvement in erythema area and selected secondary outcomes, including pore index, compared with IPL alone. While IPL primarily targets hemoglobin to induce selective photothermolysis of superficial vessels, adjunctive SSA may contribute through complementary pathways. SSA peeling has been reported to improve rosacea‐related inflammatory features and skin surface texture, potentially by modulating keratinization, reducing follicular plugging, and improving barrier‐compatible desquamation [9]. Combination strategies pairing vascular lasers with SSA‐based peeling have also been explored, supporting the concept that a keratolytic/anti‐inflammatory adjunct may enhance overall facial outcomes in rosacea beyond vascular clearance alone [10]. Other adjunctive combination regimens, such as pulsed dye laser paired with topical anti‐inflammatory therapy (e.g., topical ivermectin), have also been evaluated in rosacea [12]. In our practice, SSA was used as an adjunct to IPL in a pragmatic, patient‐centered manner. Therefore, the observed between‐group differences should be interpreted as comparative effects of the two treatment strategies rather than as evidence of an SSA‐specific effect or pharmacologic synergy. The apparent distinction between transient procedural irritation and medium‐term symptom improvement also warrants emphasis. Disease‐related stinging NRS was assessed at the 3‐month follow‐up and improved in both groups, whereas treatment‐related stinging in the safety questionnaire referred specifically to reactions occurring within 1 week after the final procedure. Thus, greater immediate sensory irritation after SSA peeling would not necessarily contradict subsequent improvement in rosacea‐associated stinging. Nevertheless, because the between‐group difference in disease‐related stinging improvement did not remain significant after FDR adjustment, this temporal interpretation remains hypothesis‐generating.

The interpretation of between‐group efficacy signals should also consider the multiplicity of outcomes. Because this exploratory real‐world study evaluated several correlated imaging, clinician‐reported, and patient‐reported end points, isolated borderline *p* values may be vulnerable to Type I error. In the FDR‐adjusted sensitivity analysis, the more robust signals were observed for erythema area, hyperpigmentation‐related parameters, pore index, RosaQoL, and GAIS distribution. By contrast, between‐group differences in erythema concentration, CEA, self‐assessed facial redness, flushing severity, and stinging NRS were not robust after age adjustment and/or FDR correction and should therefore be interpreted cautiously. Therefore, the present findings are best interpreted as hypothesis‐generating real‐world evidence supporting further prospective evaluation of SSA + IPL, rather than as confirmatory proof of superiority across all clinical domains.

The prominent improvement in hyperpigmentation‐related parameters should be interpreted in the context of treatment modality and patient‐perceived global facial improvement rather than as a defining ETR‐predominant rosacea disease endpoint. IPL can affect both oxyhemoglobin and melanin depending on wavelength selection, and SSA may enhance epidermal turnover and superficial pigment dispersion. Therefore, improvement in hyperpigmentation metrics may reflect broader dyschromia and skin‐tone benefits of the combined regimen. However, because ETR‐predominant rosacea is primarily a vascular phenotype, erythema‐related indices and CEA remain the most disease‐specific outcomes, whereas pigmentation outcomes should be regarded as exploratory adjunctive end points.

The pragmatic three‐session treatment course also reflects routine‐care decision‐making. Previous rosacea IPL studies have demonstrated clinically meaningful improvement after a limited number of sessions, whereas longer repeated IPL regimens are often designed for broader photorejuvenation goals rather than telangiectasia‐oriented vascular end points [4, 13, 14, 15, 16]. In our cohort, treatment was generally discontinued once visible improvement was considered satisfactory by both physician and patient, which may enhance external validity for cost‐ and burden‐sensitive routine practice.

Methodologically, a strength of our study is the use of objective imaging‐derived outcomes alongside clinical and patient‐reported measures. VISIA provides standardized facial imaging, and ImageJ‐based quantification adds transparency and reproducibility to erythema/pigmentation assessment [17, 18]. This multimodal design helps mitigate sole reliance on subjective grading, which is particularly relevant in ETR‐predominant rosacea where redness intensity can fluctuate with environment, stress, and daily triggers. Moreover, incorporating QoL (RosaQoL) and GAIS captures the patient‐relevant dimension of improvement that is increasingly emphasized in dermatologic outcomes research [19, 20]. Although the between‐group difference in erythema area was statistically robust, an established minimal clinically important difference for VISIA/ImageJ‐derived erythema area is not available. Therefore, the magnitude of this difference should not be interpreted as meeting a validated threshold for clinical importance, and its clinical relevance should instead be considered alongside clinician‐ and patient‐reported outcomes.

A more clinically informative distinction emerged between AE incidence and severity. Erythema and edema occurred in all patients, but edema was more intense after SSA + IPL in the unadjusted ordinal analysis. Moderate stinging was also observed only in the combination group, although the overall severity difference did not reach conventional statistical significance. These findings are biologically plausible because IPL was delivered immediately after 30% SSA peeling, when transient barrier perturbation and increased cutaneous reactivity may contribute to greater procedure‐related irritation and sensory discomfort. Importantly, no Grade 3 reactions occurred, and the edema and stinging severity signals did not remain statistically significant after FDR adjustment. They should therefore be interpreted as exploratory tolerability signals rather than definitive evidence of increased harm. For patient counseling, these findings support discussing the possibility of greater short‐term procedural irritation with the combination regimen while avoiding the implication of a definitively increased overall AE risk. Future prospective studies should separately record AE incidence, peak severity, duration, time to resolution, and treatment requirements.

Several limitations should be considered. First, the retrospective, non‐randomized design is the most important limitation. Treatment selection was based on patient preference after routine counseling, creating the potential for selection bias and residual confounding by unmeasured factors such as treatment expectations, willingness to tolerate short‐term reactions, disease perception, and adherence to skincare or trigger avoidance. In addition, a substantial proportion of otherwise eligible patients were excluded after treatment selection because of incomplete clinical data or irregular follow‐up, which may have introduced attrition bias. Although consecutive screening, uniform eligibility criteria, standardized imaging, and identical follow‐up timing were used to reduce observable heterogeneity, these measures cannot eliminate confounding inherent to the observational design. The SSA + IPL group was also significantly older than the IPL group. After adjustment for age, the between‐group difference in erythema area remained significant, whereas that in erythema concentration was attenuated, indicating that some unadjusted efficacy signals were sensitive to baseline imbalance. A sensitivity analysis excluding patients with concomitant baseline papules yielded broadly consistent findings for the principal imaging outcomes, particularly erythema area; however, the smaller subgroup limits definitive inference regarding patients with strictly isolated vascular disease. Finally, because the study compared SSA + IPL with IPL monotherapy without an SSA‐alone arm, the observed between‐group differences cannot be attributed specifically to SSA or interpreted as evidence of pharmacologic synergy. Accordingly, these findings represent exploratory comparative associations between two treatment strategies rather than causal estimates of an SSA‐specific treatment effect. Second, the modest sample size and multiplicity of outcomes limit statistical precision. Because this was a retrospective study based on an available clinical cohort, no a priori sample‐size calculation was performed, and no single primary endpoint was prospectively prespecified. Multiple objective, clinician‐reported, patient‐reported, and safety outcomes were evaluated, increasing the possibility of chance findings. Although FDR adjustment was applied as a sensitivity analysis, borderline associations—particularly those for self‐assessed redness, CEA, and stinging NRS—should be interpreted cautiously. The wide confidence interval around the age‐adjusted GAIS odds ratio also reflects limited precision despite the strong direction of association. Future prospective studies should define a limited number of primary and key secondary end points a priori and determine sample size accordingly. Third, safety data were collected retrospectively from a patient‐reported mobile questionnaire limited to the first week after the final treatment session. Moreover, the 1‐week reporting window may have failed to capture delayed or late‐onset adverse events. Although this approach captured patient‐perceived reactions, it was not equivalent to prospective AE monitoring, and detailed information on onset, duration, time to resolution, supportive treatment, and impact on daily activities was not uniformly available. The modified 0–3 severity scale also provides less granularity than prospective AE diaries or standardized clinician assessment. Consequently, the observed edema and stinging severity patterns should be regarded as exploratory tolerability signals and require confirmation using prospectively defined safety end points. Fourth, follow‐up was limited to a single assessment 3 months after the final treatment. Although this interval extends beyond immediate post‐procedural effects, it does not permit characterization of response trajectories, recurrence, or the durability of between‐group differences. Longer prospective follow‐up with repeated assessments is needed to determine whether the observed benefits persist and to inform retreatment or maintenance strategies. The importance of longer observation is supported by prospective evidence showing that recurrence can be meaningfully evaluated after IPL treatment [21]. Fifth, individualized IPL parameters reflected routine clinical practice but introduced treatment heterogeneity that may have influenced effect estimates. We attempted to improve transparency by providing the parameter ranges and treatment logic used in practice (Table S2), but this does not substitute for a standardized protocol. In addition, the single‐center setting and predominantly female, Fitzpatrick skin Type III cohort may limit generalizability to other populations, treatment settings, and skin phototypes. Multicenter prospective studies using standardized treatment protocols and stratification by phenotype and skin type are warranted.

Taken together, these findings suggest that SSA + IPL warrants further prospective evaluation as a combination strategy for patients with ETR‐predominant rosacea, particularly when erythema improvement is sought alongside broader facial outcomes such as pore‐related texture. Any potential incremental benefit should be weighed against the possibility of greater transient procedural irritation.

## 5 Conclusion

In this retrospective real‐world cohort of patients with ETR‐predominant rosacea, SSA + IPL was associated with greater improvement in erythema area and selected secondary outcomes, including hyperpigmentation‐related measures, pore index, RosaQoL, and GAIS, compared with IPL monotherapy. The association with erythema area remained robust after adjustment for age and exclusion of patients with baseline papules, whereas not all between‐group differences were consistently maintained across sensitivity analyses. Overall short‐term AE incidence was comparable between groups, although exploratory severity analyses suggested greater transient procedural irritation with combination treatment. These findings provide hypothesis‐generating comparative evidence for SSA + IPL and warrant confirmation in adequately powered prospective controlled studies with longer follow‐up.

## Author Contributions

Birao Fan: conceptualization, methodology, investigation, data curation, formal analysis, visualization, writing – original draft, writing – review and editing. Ruixing Yu: conceptualization, methodology, investigation, validation, supervision, writing – review and editing. Both authors contributed to clinical outcome assessment and approved the final manuscript.

## Funding

Supported by the National Natural Science Foundation of China (No. 82574002), Elite Medical Professionals Project of China‐Japan Friendship Hospital (No. ZRJY2024‐QMPY03), Key Project of the Capacity Building and Continuing Education Center (Party School of the National Health Commission) (No. GWJJMB202510025047), and the National High Level Hospital Clinical Research Funding (No. 2025‐NHLHCRF‐YXHZ‐MS‐03).

## Disclosure

Study registration: This retrospective observational study was not prospectively registered.

Patient and public involvement: Patients and members of the public were not involved in the design, conduct, reporting, or dissemination planning of this study.

## Ethics Statement

Ethical approval was obtained from the Institutional Ethics Committee of China‐Japan Friendship Hospital (Approval No. 2026‐KY‐370). All procedures followed the ethical standards of the Declaration of Helsinki.

## Consent

The written informed consent for photographs and/or video images was signed by all subjects.

## Conflicts of Interest

The authors declare no conflicts of interest.

## Supporting information


**Figure S1:** Flow diagram of patient enrollment. ETR‐predominant rosacea, erythematotelangiectatic‐predominant rosacea; SSA, supramolecular salicylic acid; IPL, intense pulsed light.
**Table S1:** Eligibility criteria for the study cohort.
**Table S2:** Typical IPL treatment parameters for erythematotelangiectatic‐predominant rosacea in this study.
**Table S3:** Inter‐ and intra‐rater reliability of ImageJ‐based quantitative measurements.
**Table S4:** Age‐adjusted between‐group analyses of efficacy outcomes.
**Table S5:** FDR‐adjusted sensitivity analysis for between‐group comparisons.
**Table S6:** Sensitivity analysis restricted to patients without baseline papules.

## Data Availability

The data that support the findings of this study are available from the corresponding author upon reasonable request, subject to applicable ethical and institutional requirements.
