# Supplementary material for: Efficacy and Safety of Supramolecular Salicylic Acid Peeling Combined With Intense Pulsed Light Versus Intense Pulsed Light Monotherapy in Erythematotelangiectatic‐Predominant Rosacea: A Retrospective Real‐World Comparative Study
Source: J Cosmet Dermatol. 2026 Oct 16;25(10):e71249. doi: 10.1111/jocd.71249 (PMC13675506; doi:10.1111/jocd.71249)
Supplement: Supplementary file 1 [file JOCD-25-e71249-s001.docx]

**Supplementary Materials**

**Supplementary Figure S1. Flow diagram of patient enrollment.** ETR-predominant rosacea, erythematotelangiectatic-predominant rosacea; SSA, supramolecular salicylic acid; IPL, intense pulsed light.

**Supplementary Methods**

IPL settings were individualized to balance vascular targeting and epidermal safety. Lower cut-off filters (560–590 nm) were preferentially used for erythema-oriented treatment, whereas higher cut-off filters (640–695 nm) with conservative fluence were selected for darker skin types or higher pigment risk to reduce non-specific epidermal melanin absorption; split-pulse delivery with inter-pulse delays was applied to facilitate epidermal cooling and improve tolerability.

Supplementary Table S1. Eligibility criteria for the study cohort

| **Category** | **Criterion** |
| --- | --- |
| Inclusion | Adults aged 18–65 years |
|  | ETR-predominant rosacea, defined by persistent centrofacial erythema, with or without telangiectasia, as the dominant phenotype and primary treatment target |
|  | Received SSA+IPL or IPL monotherapy according to routine-care preference |
|  | Completed at least 3 treatment sessions |
|  | Available baseline and 3-month post-treatment clinical and imaging data |
| Exclusion | Papulopustular-predominant rosacea |
|  | Baseline pustules or severe inflammatory flare |
|  | Acne vulgaris-dominant facial lesions |
|  | Other facial dermatoses affecting erythema assessment |
|  | Pregnancy or lactation |
|  | Irregular or <3-month oral tetracycline or topical rosacea treatment before enrollment |
|  | Facial energy-based procedures within the preceding 3 months |
|  | Irregular follow-up or incomplete data preventing outcome assessment |

**Supplementary Table S2. Typical IPL treatment parameters for erythematotelangiectatic-predominant rosacea in this study**

| Item | Typical range / setting | Notes (how applied in practice) |
| --- | --- | --- |
| Device | Lumenis M22 (Lumenis Ltd., Yokneam, Israel) | IPL system used for all sessions |
| Clinical target | Persistent facial erythema / telangiectatic component in ETR-predominant rosacea | Parameters individualized by erythema pattern and Fitzpatrick skin type |
| Cut-off filters (erythema-focused) | 560–590 nm | Used for erythema-oriented treatments |
| Cut-off filters (higher pigment risk) | 640–695 nm | Used for darker skin types or higher pigment-risk patients with conservative settings |
| Fluence | 10–18 J/cm² | Adjusted to tolerance and clinical endpoint |
| Pulse structure | 2–3 subpulses | Split-pulse approach to reduce epidermal overheating |
| Subpulse duration | 3.0–6.0 ms | Selected according to skin type and erythema severity |
| Interpulse delay | 10–30 ms | Adjusted to allow epidermal cooling between subpulses |
| Passes | 1–2 passes | Limited overlap to reduce excessive thermal accumulation |
| Coupling medium | Coupling gel | Applied throughout IPL procedure |
| Cooling | Contact cooling | Used throughout treatment; additional cooling post-treatment if needed |
| Session interval | 4–6 weeks | Standard interval between sessions |
| Number of sessions | 3 sessions | A fourth session could be considered for clinically meaningful residual erythema/telangiectasia, but no patient in the analyzed cohort required a fourth session. |

Supplementary Table S3. Inter- and intra-rater reliability of ImageJ-based quantitative measurements

| **Parameter** | **Inter-rater ICC (95% CI)** | **Intra-rater ICC, assessor 1 (95% CI)** | **Intra-rater ICC, assessor 2 (95% CI)** |
| --- | --- | --- | --- |
| Erythema area | 0.933 (0.875–0.963) | 0.980 (0.960–0.990) | 0.979 (0.960–0.989) |
| Erythema concentration | 0.938 (0.879–0.968) | 0.953 (0.924–0.969) | 0.947 (0.911–0.969) |
| Hyperpigmentation area | 0.924 (0.867–0.955) | 0.960 (0.926–0.978) | 0.957 (0.928–0.972) |
| Hyperpigmentation concentration | 0.943 (0.901–0.966) | 0.976 (0.961–0.985) | 0.954 (0.924–0.972) |

ICC, intraclass correlation coefficient. Inter-rater reliability was assessed using a two-way random-effects, absolute-agreement model based on the mean of three repeated ImageJ measurements. Intra-rater reliability was assessed using a two-way mixed-effects, absolute-agreement model using measurements repeated at least 2 weeks apart.

Supplementary Table S4. Age-adjusted between-group analyses of efficacy outcomes.

| Outcome | Adjusted treatment effect | 95% CI | P value | Model | Interpretation |
| --- | --- | --- | --- | --- | --- |
| Erythema area improvement | 385.66 mm² | 243.92 to 527.39 | <0.001 | Linear regression | Remained significant |
| Erythema concentration improvement | −0.06 percentage points | −0.45 to 0.34 | 0.777 | Linear regression | Not significant |
| Hyperpigmentation area improvement | 115.43 mm² | 85.02 to 145.84 | <0.001 | Linear regression | Remained significant |
| Hyperpigmentation concentration improvement | 0.69 percentage points | 0.36 to 1.03 | <0.001 | Linear regression | Remained significant |
| Wrinkle index improvement | 2.32 | −16.38 to 21.01 | 0.805 | Linear regression | Not significant |
| Pore index improvement | 43.01 | 36.60 to 49.42 | <0.001 | Linear regression | Remained significant |
| Self-assessed redness improvement | 0.47 | 0.03 to 0.91 | 0.039 | Linear regression | Nominal association; interpret cautiously |
| RosaQoL improvement | 0.79 | 0.54 to 1.03 | <0.001 | Linear regression | Remained significant |
| CEA improvement | 0.33 | −0.16 to 0.83 | 0.184 | Linear regression | Not significant |
| Flushing severity improvement | −0.11 | −0.60 to 0.38 | 0.657 | Linear regression | Not significant |
| Stinging NRS improvement | 0.62 | 0.01 to 1.22 | 0.045 | Linear regression | Nominal association; interpret cautiously |
| GAIS | OR 29.00 | 6.34 to 132.58 | <0.001 | Ordinal logistic regression | Higher GAIS category favored SSA+IPL |

Treatment group and age were included as covariates. For linear models, positive adjusted treatment effects indicate greater improvement with SSA+IPL except where the direction of the outcome is otherwise specified. For GAIS, the effect is reported as the adjusted odds ratio for being in a higher GAIS category. These analyses were added in response to the baseline age imbalance and are interpreted as sensitivity analyses in this retrospective cohort.

Supplementary Table S5. FDR-adjusted sensitivity analysis for between-group comparisons.

| **Domain** | **Outcome** | **Statistical test** | **P value** | **FDR-adjusted Q value** | **Interpretation** |
| --- | --- | --- | --- | --- | --- |
| Objective imaging | Erythema area improvement | Age-adjusted linear regression | <0.001 | <0.001 | Remained significant |
|  | Erythema concentration improvement |  | 0.777 | 0.805 | Not significant |
|  | Hyperpigmentation area improvement |  | <0.001 | <0.001 | Remained significant |
|  | Hyperpigmentation concentration improvement |  | <0.001 | <0.001 | Remained significant |
|  | Wrinkle index improvement |  | 0.805 | 0.805 | Not significant |
|  | Pore index improvement |  | <0.001 | <0.001 | Remained significant |
| Clinical | CEA improvement |  | 0.184 | 0.245 | Not significant |
|  | Self-assessed facial redness improvement |  | 0.039 | 0.066 | Not significant |
|  | Flushing severity improvement |  | 0.657 | 0.788 | Not significant |
|  | Stinging NRS improvement |  | 0.045 | 0.068 | Not significant after FDR adjustment |
|  | RosaQoL improvement |  | <0.001 | <0.001 | Remained significant |
|  | GAIS rating at 3-month follow-up | Age-adjusted ordinal logistic regression | <0.001 | <0.001 | Remained significant |
| Safety-AE incidence | Erythema | Fisher’s exact test | 1.000 | 1.000 | Not significant |
|  | Edema |  | 1.000 | 1.000 | Not significant |
|  | Pain |  | 0.599 | 0.959 | Not significant |
|  | Dryness |  | 1.000 | 1.000 | Not significant |
|  | Stinging |  | 0.775 | 1.000 | Not significant |
|  | Pustules |  | 0.575 | 1.000 | Not significant |
|  | Exudation and crust |  | 0.410 | 0.959 | Not significant |
|  | Hyperpigmentation |  | 0.293 | 0.959 | Not significant |
| Safety-AE severity | Erythema | Mann–Whitney U test | 0.071 | 0.199 | Not significant |
|  | Edema |  | 0.009 | 0.073 | Not significant after FDR adjustment |
|  | Pain |  | 0.546 | 0.624 | Not significant |
|  | Dryness |  | 0.856 | 0.856 | Not significant |
|  | Stinging |  | 0.075 | 0.199 | Not significant |
|  | Pustules |  | 0.536 | 0.624 | Not significant |
|  | Exudation and crust |  | 0.331 | 0.530 | Not significant |
|  | Hyperpigmentation |  | 0.257 | 0.513 | Not significant |

For efficacy outcomes, Benjamini–Hochberg FDR adjustment was applied to P values derived from the age-adjusted regression models. Linear regression was used for continuous efficacy outcomes, and ordinal logistic regression was used for GAIS. For safety outcomes, AE incidence and ordinal AE severity were treated as separate comparison families. Incidence P values were derived from Fisher’s exact tests, and severity P values from Mann–Whitney U tests. FDR-adjusted Q values were calculated using the Benjamini–Hochberg method.

Supplementary Table S6. Sensitivity analysis restricted to patients without baseline papules.

| Outcome | SSA+IPL (n=20), median improvement | IPL (n=25), median improvement | P value | Interpretation |
| --- | --- | --- | --- | --- |
| Erythema area, mm² | 1180.35 | 779.97 | <0.001 | Remained significant |
| Erythema concentration, percentage points | 2.215 | 1.920 | 0.059 | Not significant |
| Hyperpigmentation area, mm² | 213.10 | 112.42 | <0.001 | Remained significant |
| Hyperpigmentation concentration, percentage points | 2.105 | 1.560 | <0.001 | Remained significant |
| Wrinkle index | 66.0 | 67.0 | 0.664 | Not significant |
| Pore index | 68.5 | 29.0 | <0.001 | Remained significant |
| Self-assessed redness | 3.0 | 3.0 | 0.064 | Not significant |
| RosaQoL | 2.35 | 1.70 | <0.001 | Remained significant |
| CEA | 3.0 | 3.0 | 0.072 | Not significant |
| Flushing severity | 2.0 | 2.0 | 0.095 | Not significant |
| Stinging NRS | 2.0 | 2.0 | 0.034 | Nominal association; interpret cautiously |

Patients with any baseline papules were excluded (12 of 57 patients), leaving 45 patients. P values were calculated using the Mann–Whitney U test. This analysis was performed to assess whether the main findings were dependent on inclusion of patients with mild inflammatory components.
